# Supplementary material for: Toxic behaviour facilitates echo chammber formation: An agent-based modelling simulation of science attitudes based on Spiral of Silence Theory
Source: PLoS One. 2025 Jun 5;20(6):e0323849. doi: 10.1371/journal.pone.0323849 (PMC12140271; doi:10.1371/journal.pone.0323849)
Supplement: S1 File — Robustness checks for changes in starting networks and parameter values not reported in the paper, and the effects of changes to the definition of an echo chamber. (PDF) [file pone.0323849.s001.pdf]

Supplement

Timothy F. Bainbridge[1,*] Sinead Golley[1] Matthew Ryan[1] Naomi Kakoschke[1] Emily Brindal[1]

2025-02-20

[1] Health and Biosecurity, CSIRO, Adelaide, South Australia, Australia

[*] Correspondence: Timothy F. Bainbridge <Tim.Bainbridge@csiro.au>

Contents

1 Introduction **2**

2 Alternative Parameter Sets **2**

2.1 Network Changes 2
2.2 SD 5
2.3 Freind Costs and Benefits 5
2.4 Discount Rates 5
2.5 Payoff Differences 5

3 Echo Chamber Definition **6**

List of Figures

1 Boxplots of the percent of agents in an echo chamber for selected simulations grouped by the type of change from Base. 3
2 Boxplots of mean opinions for selected simulations grouped by the type of change from Base. 3
3 Density plots of agents' versus neighbours' opinion with overlaid individual data coloured by echo chamber membership for selected simulations grouped by the type of change from Base. 4
4 Boxplots of the different definitions of echo chambers. 6
5 A density plot of agents' and neighbours' opinions with discripancies between the standard definition of echo chambers and the exclusive definition highlighted. 7

1 Introduction

This file is the supplementary information document for the paper, "Toxic behaviour facilitates echo chamber formation: An agent-based modelling simulation of science attitudes based on Spiral of Silence Theory" submitted to Plos One.

The document contains two sections. The first section compares outputs from changes to parameters or starting conditions not explored in the paper to check the model's robustness to these parameter changes (mentioned in the "3.4 Robustness Checks" section of the paper). The second section examines how results from the paper change with different definitions of an echo chamber.

2 Alternative Parameter Sets

Changes to default parameter values were tested. The various alternatives are listed in Table 1, including a short name, the change category, a description, and the change made. For further information about the parameters, see Table 3 from the model documentation (https://doi.org/10.31234/osf.io/jr493).

The different simulations are compared in figures based upon figures from the manuscript. These include the proportions of agents who end up in an echo chamber in Figure 1, mean opinions in Figure 2, and density graphs in Figure 3.

Table 1: Descriptions of the alternative parameters values tested.

**Change Category**	**Simulation Name**	**Description**	**Simulation Changes**
Base	Base	The baseline simulations.	N/A
Network	Network 1	An alternative cluster starting network.	Different starting network.
	Network 2	A second alternative cluster starting network.	Different starting network.
	Lattice Network	A lattice starting network.	Different starting network.
	1000 Agents	A cluster starting network with 1000 agents.	Different starting network.
SD	Halved SD	Halved model variance.	$\sigma_e = 0.075, \sigma_f = 0.2$
	Doubled SD	Doubled model variance.	$\sigma_e = 0.3, \sigma_f = 0.8$
	Individual SD only	All variance moved to f_{iJI}.	$\sigma_e = 0, \sigma_f \approx 0.43$
	Grouped SD only	All variance moved to e_{jI}.	$\sigma_e \approx 0.43, \sigma_f = 0$
Friend Cost	Friend cost reduced	Cost of maintaining connections reduced.	$c_{KG} = 0.04$
	Friend cost increased	Cost of maintaining connections increased.	$c_{KG} = 0.0625$
Friend Benefit	Friend benefit reduced	Benefit of having connections halved.	$c_{BG} = 0.1$
	Friend benefit increased	Benefit of having connections doubled.	$c_{BG} = 0.4$
Discount	Discount halved	Dicount rates halved.	$d_T = 0.45, d_A = 0.45$
	Myopic	Myopic agents. Discount rates set to 0.	$d_T = d_A = 0$
Payoff Difference	Payoff difference elimiated	Difference in payoffs to post set to 0.	$u = 0$
	Payoff difference doubled	Difference in payoffs to post set to 0.4.	$u = 0.4$
	Payoff difference quadrupled	Difference in payoffs to post set to 0.8.	$u = 0.8$

2.1 Network Changes

Figures 1 to 3 reveal few differences between the baseline starting network, the alternative cluster networks (Network 1 and Network 2), the network with 1000 agents, and, surprisingly, the lattice network. The starting lattice network was not a small world network, so would be expected to result in the largest differences in outcomes if starting network structure was important. Instead, the dynamic nature of the network in PASOM seemed to allowed connections to form sufficiently quickly that results were similar to the cluster networks. Instead, differences in outcomes were greatest for the alternative cluster networks, with somewhat different

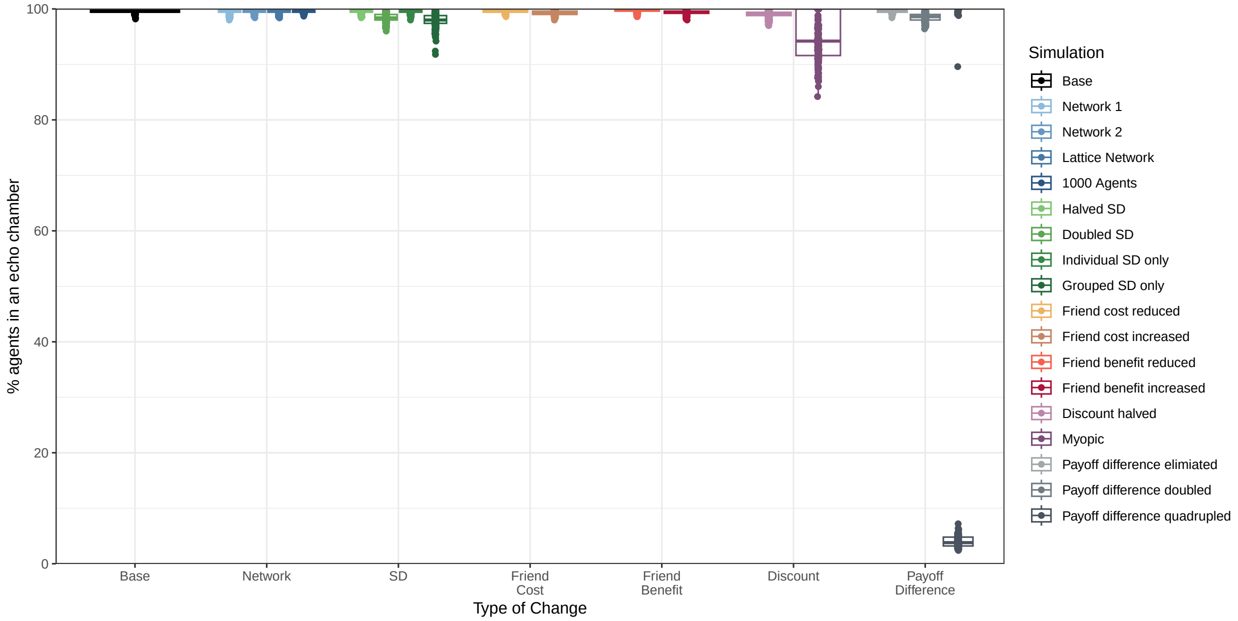

Figure 1: Boxplots of the percent of agents in an echo chamber for selected simulations grouped by the type of change from Base.

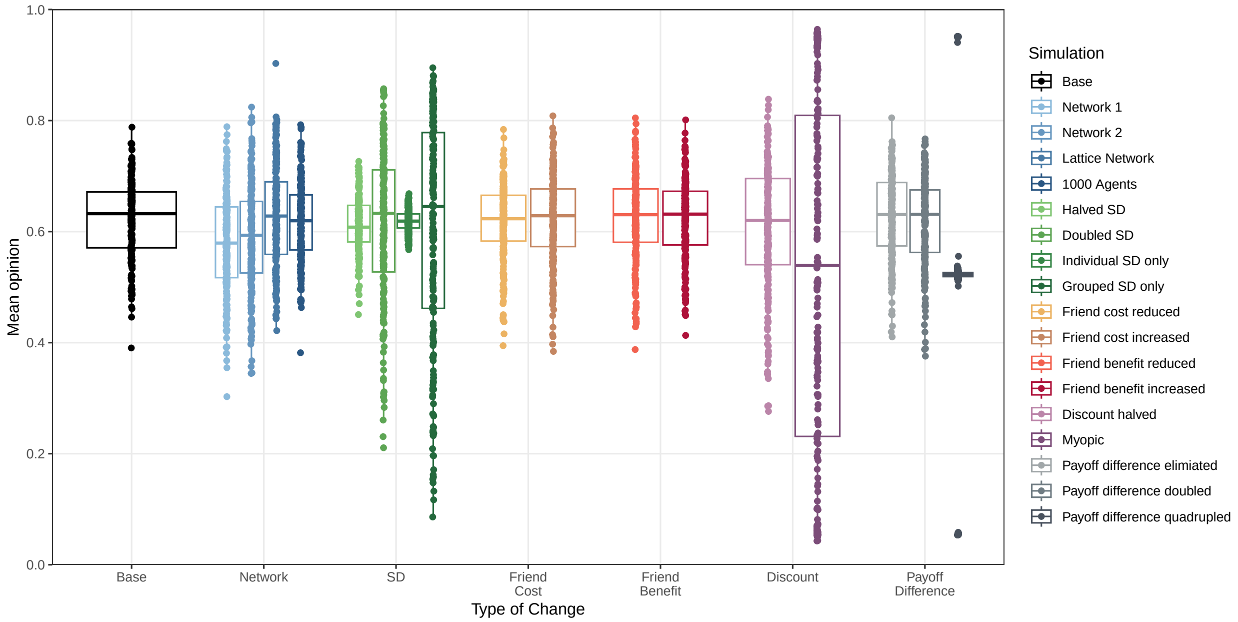

Figure 2: Boxplots of mean opinions for selected simulations grouped by the type of change from Base.

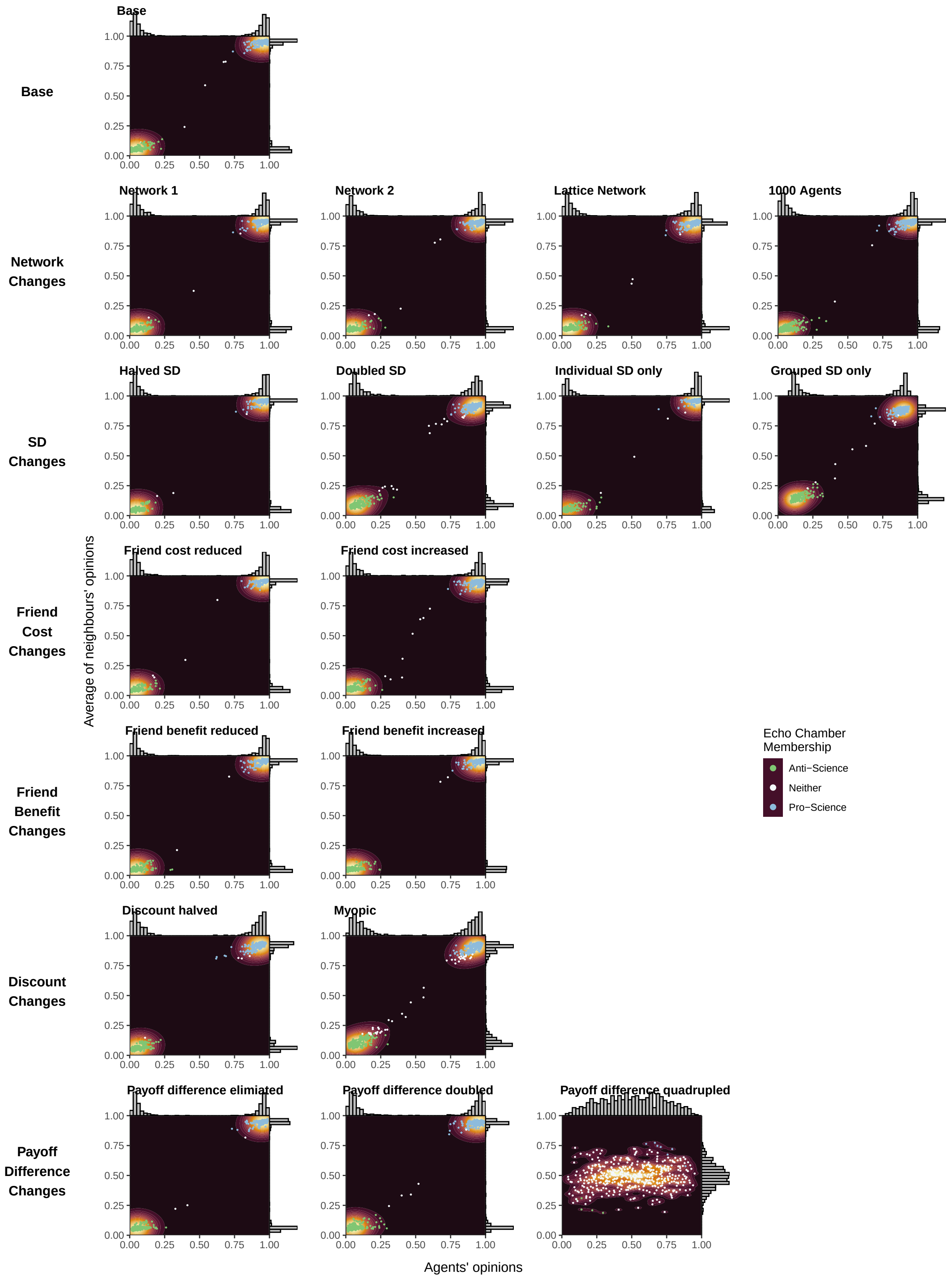

Figure 3: Density plots of agents' versus neighbours' opinion with overlaid individual data coloured by echo chamber membership for selected simulations grouped by the type of change from Base.

distributions of mean opinions, suggesting that some differences may arise from starting structures, but these are not obviously predictable from highly salient highly salient network features. Therefore, given the largest differences in outcomes were for the conceptually most similar starting networks, it follows that outcomes and conclusions were unlikely to be overly influences by the choice of the starting network structures.

2.2 SD

Two random factors were included in PASOM, e_{jI} and f_{ijI}. These variables affected the payoffs of agents from round to round, either collectively (for e_{jI}) or individually (for f_{ijI}). Simulations halved and doubled the standard deviation of these variables, made all the variance individual variance (i.e., $e_{jI} = 0$), and made all the variance group variance (i.e., $f_{ijI} = 0$). Total variance was preserved in the latter two cases.

Halved and doubled variance had the expected effects on opinions with less and more variance in mean opinions between simulations, respectively. Making all the variance individual variance substantially reduced the spread in outcomes, and making all the variance group variance substantially increased the spread of outcomes, noticeably reduced echo chamber membership (although it remained high), and reduced the distance between clusters in the density plots. The changes between the individual and group variance simulations was likely due to individual differences in f_{ijI} in general cancelled out, whereas changes in e_{jI} did not. Changes in e_{jI} would make neutral agents more likely to act similarly, potentially generating new connections, and would make extreme agents opposed to the popular view less likely to act against the position, meaning fewer connections were severed. These differences would allow more connections to persist explaining both the lower echo chamber membership and reduced distance between clusters.

2.3 Freind Costs and Benefits

Friend cost and benefit parameters (i.e., c_{KG} and c_{BG}, respectively) affect how many agents each agent wants to be connected to. Changes to these parameters made minimal difference to the outcomes. Reducing c_{KG} or increasing c_{BG} would make agents somewhat less likely to disconnect early and more likely to create new connections but after some rounds the equilibrium would be reached and typical the typical network dynamics would occur. Reversing the changes would have a similar but reversed effect, which, in either case, caused minimal changes. These parameters may matter at the extreme where agents would only desire connections in very positive environments (or not at all), but the point of the section is to explore sensible values of the parameters not edge cases.

2.4 Discount Rates

These simulations changed the rate at which agents discounted their expectations of constructive and toxic behaviour. The first halved the rate (from 0.9 to 0.45) and the second made agents myopic by reducing the discount rate to 0. That meant that agents would only consider the previous round in their calculations of the expected number of constructive and toxic posts they would see in the current round. Reductions in the discount rate changed the time frame of the 'Spiral of Silence' effect to make more recent rounds more important (or the last round the only important round) and make more distant rounds less important. Reductions in the discount rate increased the variance of mean opinions and somewhat decreased the presence of echo chambers; however, differences were quite small for the simulations where values where the discount rate was dropped to 0.45, despite this change being substantial. Therefore, only extreme changes for the parameter have major effects on simulation outcomes.

2.5 Payoff Differences

In order to decide to post, agents had to have a positive payoff and the difference between payoffs for sharing a pro- or anti-science position had to be greater than a value of the parameter, u. Changes in this parameter had relatively small effects on model outcomes until they reached a value where agents would rarely share. Thus, for the simulations where the parameter was quadrupled from the baseline value, some simulations included very little activity where very little changed from the starting situation. Alternatively, early activity by one side or the other would occasionally prompt further activity on that side due to agents' expectations of

constructive activity in their payoff functions. This dynamic created three distinct types of outcomes—almost entirely pro-science, almost entirely anti-science, or neutral—which can be seen in Figure 2. Given the neutral situation is between the two extremes, one of the neutral outcomes is displayed in Figure 3.

3 Echo Chamber Definition

To categorise agents as members of either a pro- or anti-science echo chamber, a definition of an echo chamber was required. The definition chosen required agents to have an opinion more extreme than 0.1 from the midpoint (i.e., ≥ 0.6 or ≤ 0.4) and have 90% of their connections be on the same side of the midpoint as themselves. To check that this definition did not have a substantial effect on our results compared to other reasonable definitions, we compared the results with this definition to a more exclusive definition, where agents had to have an opinion ≥ 0.75 or ≤ 0.25 and had to have at least 95% of their connections share their view, and a more inclusive definition, where agents had to have an opinion ≥ 0.6 or ≤ 0.4 (as in the standard definition) and had to have at least 75% of their connections share their view. Where outcomes were at the system level, we also compared results to a measure of homophily, calculated with the "assortivity" function from the igraph package. These comparisons were made for the baseline simulations.

Figure 4 shows the distributions of proportions of echo chamber membership for each of the different definitions tested and the homophily score (with higher numbers meaning more separate). Differences between the standard definition, the inclusive definition, and even the homophily scores were minimal with only the exclusive definition showing markedly lower rates of echo chamber membership.

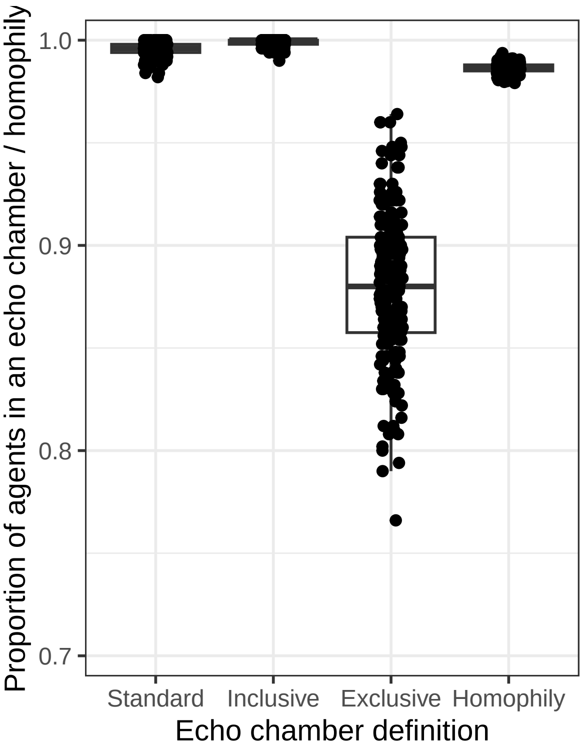

Figure 4: Boxplots of the different definitions of echo chambers.

To see the effect on individual agents between the standard and exclusive definitions, a simulation with a moderate outcome was selected and agents opinions versus the average of their neighbours opinions were plotted. The resultant figure is shown in Figure 5. In the figure, "discrepant" agents—classed as "in an echo chamber" with the standard definition and "not in an echo chamber" with the exclusive definition—are highlighted in red.

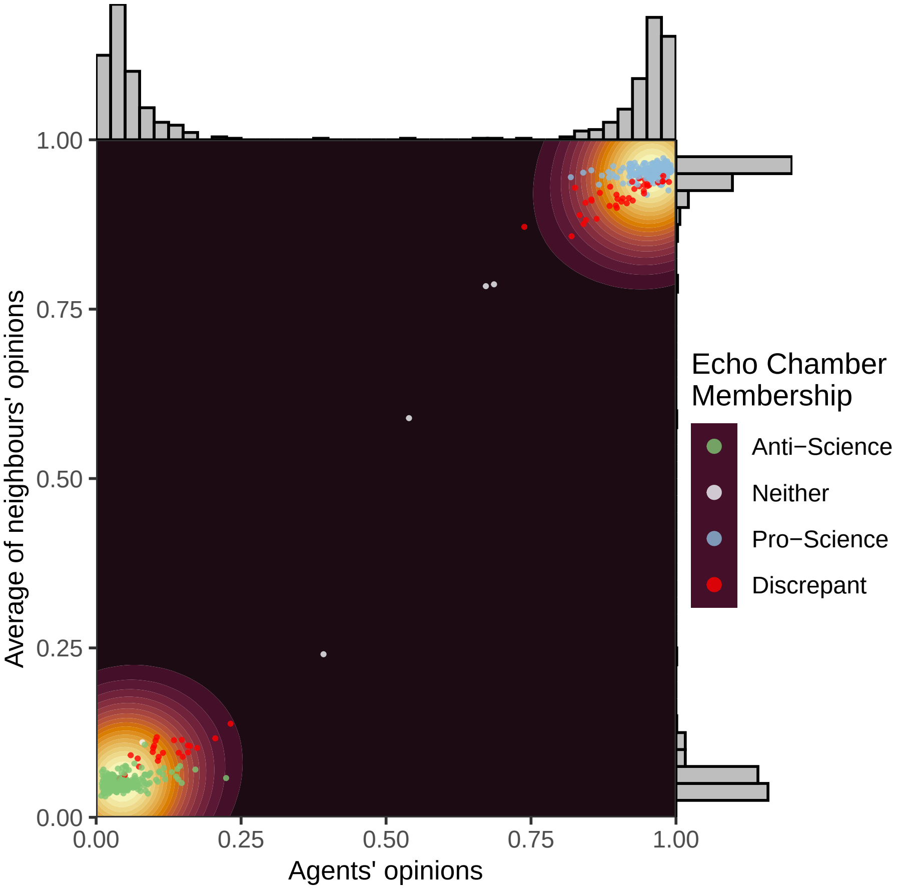

Figure 5: A density plot of agents' and neighbours' opinions with discripancies between the standard definition of echo chambers and the exclusive definition highlighted.

Two observations are worth noting from the figure. First, agents' opinions made no difference to the definitions used as all agents in an echo chamber in either definition had a opinions more extreme than the criteria used for the exclusive definition. Although agents' opinions likely would have made a difference to the progression of echo chamber membership through the rounds, they were not important at the final round (at least given the baseline starting conditions and parameters). This means that only neighbours' opinions affected differences in echo chamber membership by definition. Second, although discrepant agents tended to have less extreme neighbours than non-discrepant echo chamber members, on average, many of the discrepant agents were centrally located within the clusters representing the pro- and anti-science echo chambers. Therefore, the exclusive definition is far too exclusive a definition—at least for the current modelling purpose.

Thus, although a somewhat more or less exclusive definition of echo chamber membership may have been possible, the definition used in the paper was not unreasonable. Any definition that would have substantially changed results could only have done so by excluding agents from echo chambers who should reasonably have been included.
